# Comparative Evaluation of Dynamic Maceration and Ultrasonic Assisted Extraction of Fucoidan from Four Arctic Brown Algae on Its Antioxidant and Anticancer Properties

**DOI:** 10.3390/md23060230

**Published:** 2025-05-28

**Authors:** Ekaterina D. Obluchinskaya, Olga N. Pozharitskaya, Irina M. Lapina, Anna A. Kulminskaya, Elena V. Zhurishkina, Alexander N. Shikov

**Affiliations:** 1 Murmansk Marine Biological Institute of the Russian Academy of Sciences (MMBI RAS), 17 Vladimirskaya Str., Murmansk 183038, Russia; obluchinskaya@gmail.com (E.D.O.); olgapozhar@mail.ru (O.N.P.); 2 Petersburg Nuclear Physics Institute Named by B.P.Konstantinov of NRC “Kurchatov Institute” (NRC “Kurchatov Institute”—PNPI), 1 Orlova Roshcha Str., Leningradskaya Region, Gatchina 188300, Russia; lapina_im@pnpi.nrcki.ru (I.M.L.); kulminskaya_aa@pnpi.nrcki.ru (A.A.K.); zhurishkina_ev@pnpi.nrcki.ru (E.V.Z.); 3 Department of Technology of Pharmaceutical Formulations, St. Petersburg State Chemical Pharmaceutical University, 14a Prof. Popov Str., Saint Petersburg 197022, Russia

**Keywords:** fucoidan, phlorotannins, paclitaxel, brown algae, extraction, activity, synergy

## Abstract

The technology of fucoidan extraction significantly affects its properties. This study aimed to evaluate the impact of dynamic maceration (DM) and ultrasound-assisted extraction (UAE) on the antioxidant and anticancer properties of fucoidan from Arctic brown algae. *Fucus vesiculosus* (Fv), *Fucus serratus* (Fs), *Fucus distichus* (Fd), and *Ascophyllum nodosum* (An) were collected from the Barents Sea. The average yield of fucoidan and uronic acid was higher (by 43.2% and 22.0%, respectively) after UAE, while phlorotannin content decreased by 53.7% compared with DM. The fucose level for all algae increased after UAE, while the molecular weight of fucoidans was lower. The highest antioxidant activity was noted for the fucoidan from An and Fv, which were obtained by DM and can be associated with the high concentrations of phlorotannins. The treatment of HeLa G-63 cells with all studied fucoidans for 48 h increased concentration-dependently the number of dead cells. The most promising were Fv and Fs fucoidans with high phlorotannins, low sulfates, and uronic acid extracted by DM. The co-administration of paclitaxel and fucoidan caused cell cycle arrest in the G2/M phase. The calculated for the first time combinatory effect showed that the simultaneous use of paclitaxel and fucoidan exposure leads to a synergistic interaction. Our results support the rationality of fucoidan use in complex chemotherapy to improve survival, quality of life and immunity in patients with cervical carcinoma.

## 1. Introduction

Life originated in the seas and oceans with a vibrant biodiversity, covering more than 70% of the Earth’s surface. The marine environment contains several species of plants, animals, and microorganisms producing various natural products [1]. Arctic brown algae are a perspective source of unique compounds that can create various products with beneficial properties. In the Northern Hemisphere, the genera Fucus, Ascophyllum, and Pelvetia of the family Fucaceae predominate in the intertidal zones of many cold and warm temperate regions [2,3]. These algae are the best known and are of increasing importance due to their high content of various phytochemicals with industrial applications [4,5,6].

Various species of brown algae have traditionally been used as food or dietary supplements in people’s daily diets, typically in Far East Asian countries where seaweed consumption is part of their culture. In addition, although less frequently, some species of Fucus have also been consumed in coastal countries of Western Europe, the Arctic region, and Alaska [7]. Long-term scientific studies on patients have revealed correlations between a diet containing algae and a reduced risk of developing arterial hypertension, coronary heart disease, and cerebrovascular and oncological diseases [8,9,10]. It has been shown that including algae in the diets of patients with cardiovascular diseases reduces the risk of death from complications [11]. In the case of patients with oncological diseases, it helps to increase life expectancy [12]. Expanding interest in algae as a wealthy source of bioactive metabolites (primarily polysaccharides) and macro- and micro-elements has revealed promising applications in functional foods and nutraceutical manufacturing [13,14].

The major sulfated polysaccharide known as fucoidan is extracted from marine brown algae. The structure of fucoidan is heterogeneous and varies significantly in composition and chemical structure between species, depending on both regional and seasonal influences and the extraction method, enzymatic structural modifications, and purification [15,16,17]. Fucoidan consists mainly of *L*-fucose and sulfate [18,19,20]. It also comprises other monosaccharides like galactose, glucose, mannose, rhamnose, arabinose and xylose, and uronic acids [21,22].

Fucoidan has been reported to have antioxidant, antiviral, antibacterial, anti-inflammatory, anticoagulant, antiobesity activities, etc. [23,24,25]. Accumulated evidence supports the notion that the use of fucoidan as a supplement offers protection against various types of cancer. Some authors have reported that fucoidan has antiangiogenic and antiproliferative activity against cancer cells in vitro, as well as inhibitory activity against tumor growth in mice [26]. Fucoidan has been shown to exhibit antimetastatic activity by blocking the interaction between cancer cells and the basement membrane. Fucoidan inhibits cancer cells proliferation and cancer cells adhesion to various substrates. Several in vitro and in vivo studies have shown that fucoidan has strong antitumor activity [27,28]. Fucoidans have been shown to have low toxicity to normal cells [29,30]. Since fucoidan also has an immunomodulatory effect [31], it has been postulated that it may have a protective effect against the development of side effects when combined with chemotherapeutic agents and radiation [32]. These studies showed the potency of fucoidan as a chemopreventive agent and are the basis for the effort to develop fucoidan as a chemotherapeutic agent to increase the cytotoxic activity and reduce the side effects of paclitaxel.

Diverse methods have been described for fucoidan extraction, ranging from traditional maceration to modern and advanced. According to the data discussed in a recent review [33], the yield of fucoidan varied from 2.26% (hot water maceration from *Sargassum vulgare*) up to 19.5% (extraction by McIlvaine’s buffer solution at 60 °C and pH 4.0 from Sargassum spp.). In some studies, microwave-assisted extraction was established as an effective method for the extraction yield [34], while other authors have advocated ultrasound-assisted extraction [35].

In some publications, polyphenolics coextracted with fucoidan are considered as contaminants [24]. The brown color of the algae and, consequently, of the crude fucoidan, is primarily due to polyphenols (e.g., phlorotannins) and fucoxanthin, which are closely coupled with fucoidans in the cell wall [22]. Breaking this bond requires significant purification expenses. However, some authors have shown that polyphenolic impurities in fucoidan contribute to antioxidant, anti-inflammatory, anticancer, and other activities [15,34,36].

Scientific teams from various countries have attempted to link the activities with fucoidan’s structural features, including molecular weight, monomer composition, sulfate content, and sulfation pattern. Nevertheless, this structure-activity relationship is still not well verified and varies depending on the extraction methods used [37,38,39,40].

However, the systematic comparison of the effects of extraction methods on the antioxidant and anticancer activities of fucoidans isolated from different brown algae species has not been performed yet. Although synergy for fucoidan with some anticancer drugs was claimed in the literature, the synergistic effect was not calculated.

The aim of this study was (a) to compare the efficacy of dynamic maceration (DM) and ultrasound-assisted extraction (UAE) of fucoidan from four Arctic brown alga species, (b) to evaluate the antioxidant activity and cytotoxicity of isolated fucoidans on HeLa G-63 cells, and (c) to study synergistic effect of fucoidan with paclitaxel (Ptx) on inhibition of HeLa G-63 cells.

## 2. Results and Discussion

### 2.1. The Impact of Extraction Methods on the Yield and of Fucoidans Composition

**Figure 1:**
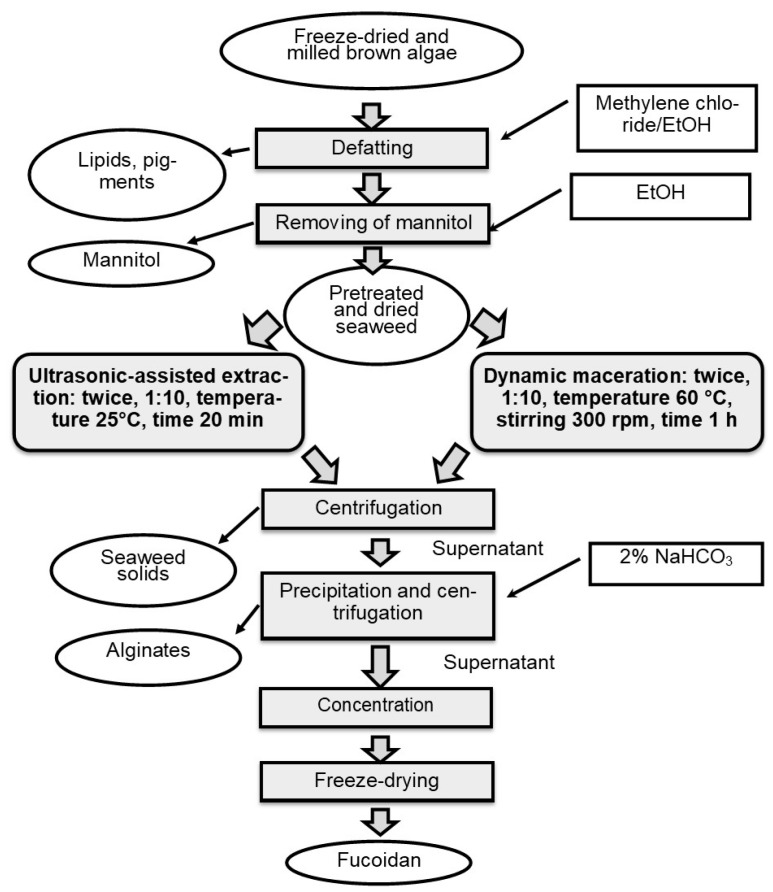
Scheme of extraction brown algae using dynamic maceration and ultrasound-assisted extraction.

The fucoidans analyzed in this study are native, unfractionated polysaccharides, which contain other co-extracted substances such as polyphenols and uronic acids. Therefore, the term “fucoidan yield” is used rather than “sulfated polysaccharide yield” [24]. The effectiveness of two intensive methods (DM and UAE) for the extraction of fucoidan was compared in this study (Figure 1).

The DM combines maceration with mechanical homogenization and temperature. In this case, a forced convection phenomenon allows a more efficient extraction in less time [41]. UAE was used to obtain polysaccharides from algae [42] and can be applied simultaneously to acid extraction [43] or as a previous pretreatment before acid extraction [44]. The effect of two different extraction methods on fucoidan yield is shown in Table 1.

**Figure 2:**
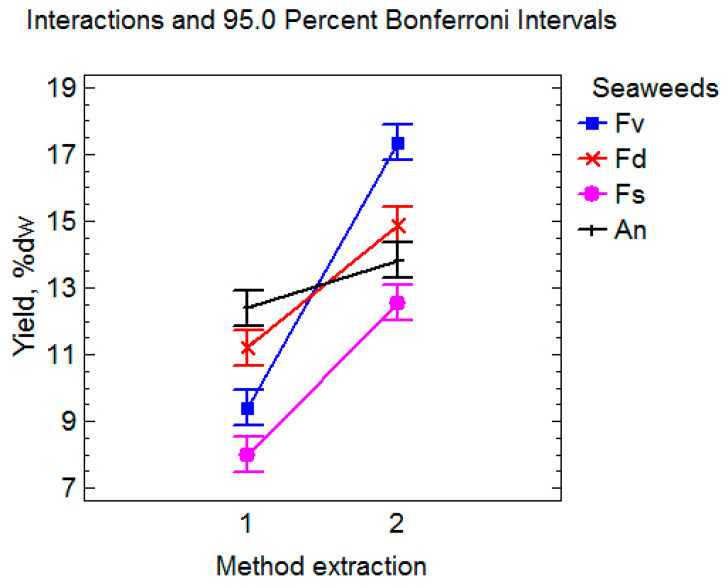
Effect of extraction method on fucoidan yield: 1—dynamic maceration and 2—ultrasound-assisted extraction.

**Figure 3:**
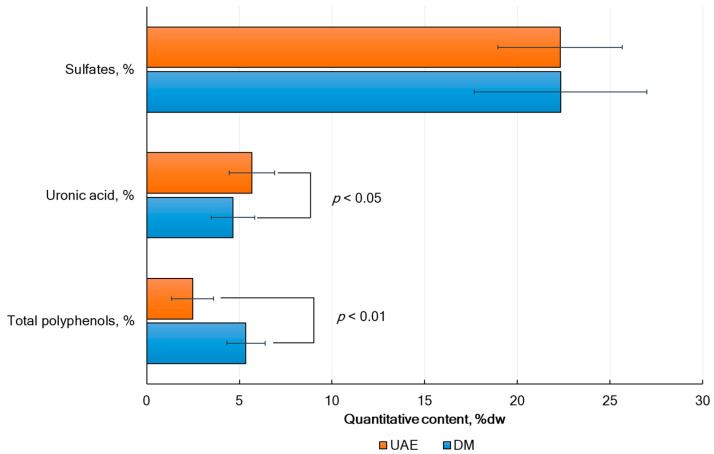
Impact of method extraction on biochemical composition of fucoidans. Data are shown as a mean ± sd (*n* = 3) for four types of algae. Multiple comparison analyses show statistically significant differences between DM and UAE extraction methods.

It was found that the extraction method affects not only the yield of fucoidan and its composition, including sulfates, uronic acids, and total phlorotannins, but also the monosaccharide composition and molecular weight of the polysaccharide (Table 1 and Table 2). When using UAE (mean for four algae species), a significant increase in fucoidan yield by 43.2% (Figure 2) and uronic acid levels by 22.0% (Figure 3) was observed. At the same time, the content of total phlorotannins was (on average) significantly lower by 53.7% than with DM (Figure 2). A two-way ANOVA analysis of the results revealed an extremely significant interaction between the extraction method and species of algae (*p* < 0.0001). The interaction of these factors was also statistically significant (Figure 2).

It is known that fucoidan fractions with similar structural characteristics often exhibit identical biological activity [45]. All fucoidans obtained by two technologies from the studied algae contained fucose as the main monosaccharide, galactose, mannose, xylose, glucose, uronic acid residues, and sulfate and were, thus, heteropolysaccharides [46]. The changes in the monosaccharide composition of fucoidan were detected after the exposure of algae material to low-frequency ultrasound. The fucose level for all algae significantly increased (Table 2), while a tendency to decrease in other monosaccharides, such as xylose, mannose, and glucose, was observed. The content of uronic acid residues and sulfates also changed under the influence of ultrasound for each algae species (Table 2).

**Figure 4:**
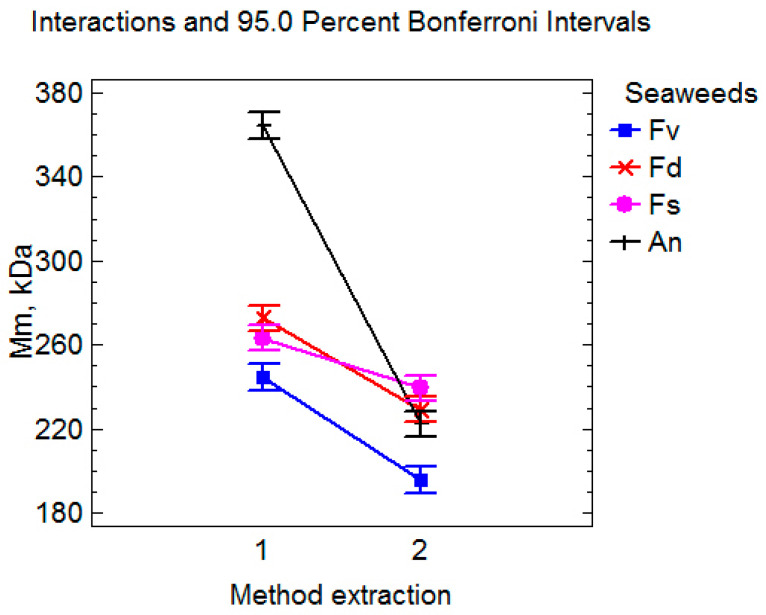
Effect of extraction method on the mean molecular weight of fucoidan. 1—dynamic maceration and 2—ultrasound-assisted extraction.

The influence of the extraction procedure conditions on the molecular weight of fucoidan is shown in Figure 4.

The molecular weight of all fucoidan samples after extraction in ultrasound was statistically lower than after extraction by the dynamic maceration method. It has been previously reported that high-molecular-weight polymers with long chains are preferentially destroyed under the action of shear forces arising from the rapid collapse of cavitation bubbles [47,48].

### 2.2. The Antioxidant Activities of Fucoidans

**Figure 5:**
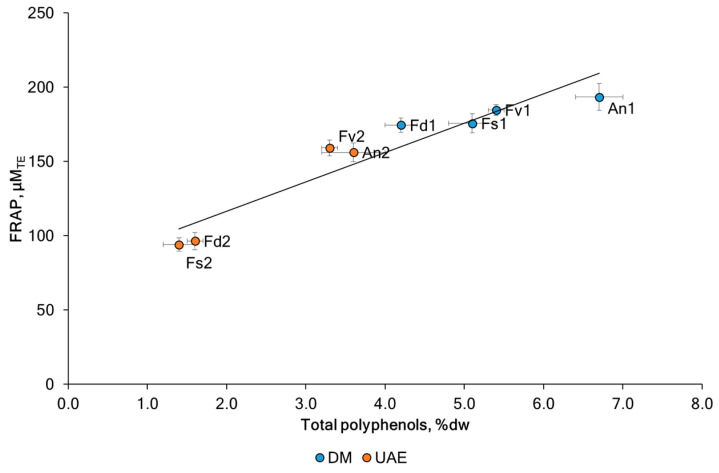
The antioxidant activity (FRAP) in Trolox equivalents and total phlorotannins content (TPC) in (phloroglucinol equivalents/g DW) for fucoidans extracted from the tested brown seaweeds extracted by DM and UAE: *F. vesiculosus* (Fv), *F. distichus* (Fd), *F. serratus* (Fs), and *A. nodosum* (An). Values are expressed as the mean ± sd.

The antioxidant potential of algae makes them interesting candidates for pharmacological, food, and medicinal applications. The ferric reducing antioxidant power (FRAP) assay was conducted on the fucoidans to study the effect of the method intensification strategy. The antioxidant capacity of fucoidan fractions is presented in Table 1. The analysis of the FRAP values shown in Figure 5 shows significant differences between the species and between the extraction methods used. The highest FRAP activity was noted for the fucoidan An1 (193 ± 9 μM_TE_) and Fv1 (185 ± 4 μM_TE_) which can again be attributed to the presence of high TPC in the same samples (6.7 ± 0.3 %dw and 5.4 ± 0.1 %dw). The FRAP value of the fucoidan extracted with UAE was decreased by 31.1% (from 13.8% for Fv to 46.5% for Fs) for fucoidan obtained by DM. These results are in line with recent findings [48,49,50,51] and support the assumption that rather co-extracted phenolic compounds (e.g., phlorotannins) and terpenoids (e.g., fucoxanthin) than the fucoidan itself exhibit antioxidant effects. In the former studies, the radical scavenging potential of fucoidans correlated with their total phenolic content and fell after purification by ion exchange chromatography. A similar effect was achieved by simply incubating *F. vesiculosus* fucoidan with H_2_O_2_, which destroyed most of the brown-colored co-extracted polyphenols and terpenoids and, thus, also reduced its antioxidant activity [51]. It was noted that the fraction with the highest fucoidan content (*p* < 0.05) had the least antioxidant activity [52]. The influence of different extraction methods, including ultrasound, was discussed in [53]. The authors showed that the FRAP activity for fucoidan samples of brown macroalgae (*Padina tetrastromatica* and *Turbinaria conoides*) obtained using ultrasound was lower than the conventional approach. A strong positive correlation was observed between FRAP and TPC (Pearson correlation coefficient r = 0.95, *p* < 0.05). Our results are consistent with previous studies reporting a positive correlation between FRAP and polyphenolic compounds for seaweed extracts [15,54]. We observed a negative correlation between FRAP levels and sulfates (Pearson correlation coefficient r = −0.26, *p* < 0.05) and uronic acid (Pearson correlation coefficient r = −0.47, *p* < 0.05). It was suggested that this was associated with phlorotannins and sulfates.

### 2.3. Anticancer Properties of Fucoidans

**Figure 6:**
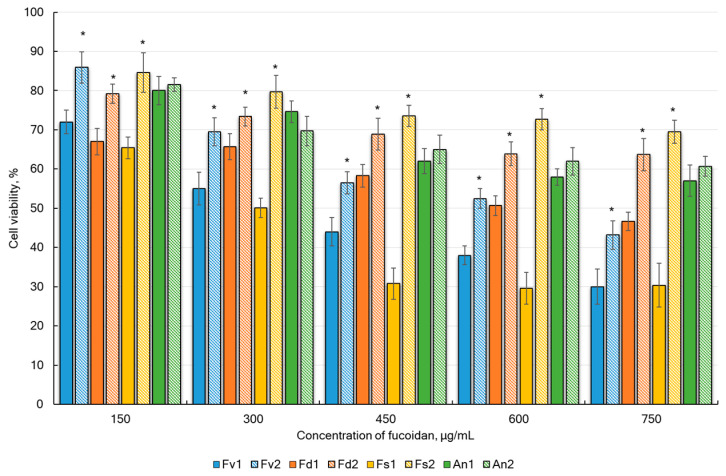
Dose-response of HeLa G-63 cell line treated with four different fucoidans extracted via DM and UAE in concentrations ranging from 150 µg/mL to 750 µg/mL: *F. vesiculosus* (Fv), *F. distichus* (Fd), *F. serratus* (Fs), and *A. nodosum* (An). The relative cell viability was calculated in comparison with the untreated negative control. * Multiple comparison analyses show statistically significant differences (*p* < 0.05) between DM and UAE extraction methods.

In the present study, the anticancer activities of fucoidans extracted from *F. vesiculosus*, *F. distichus*, *F. serratus*, and *A. nodosum* via DM and UAE were tested at five concentrations ranging from 150 to 750 µg/mL. According to the MTT test, treatment of HeLa G-63 cells with all studied fucoidans for 48 h increased the number of dead cells depending on the concentration of sulfated polysaccharides (Figure 6). It was found that the extraction method significantly affected the anticancer activity. A decrease in activity was observed under the action of ultrasound. UAE had a less pronounced effect on *A. nodosum*. No difference in the anticancer activity was observed for the fucoidan from this alga.

The anticancer properties of fucoidan have been studied since the 1980s. Further experiments have shown that these molecules can suppress cancer cell proliferation, induce apoptosis, suppress angiogenesis, etc., in vitro and in vivo studies [55,56]. According to literature data, fucoidan molecular weight has been considered one of the important factors that affect in vitro anticancer activities. Low molecular weight has been reported to have higher cytotoxicity due to its ability to increase solubility and penetration into the cell with a reduced toxic effect on normal cells [29,30]. An anticancer study using fucoidan fractions against the growth of U-251MG glioblastoma multiforme cancer cells demonstrated previously that the >300 kDa fraction had the lowest IC_50_ value against tumor cells compared to the other four fractions with a molecular weight of <300 kDa [57].

**Figure 7:**
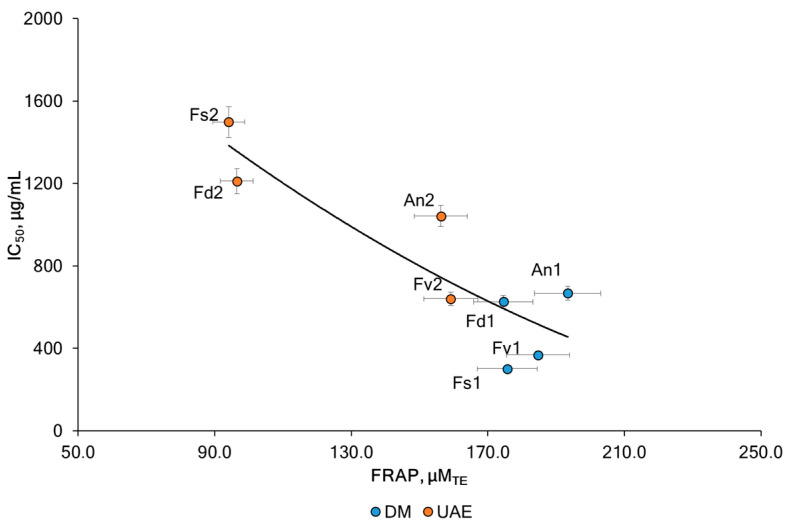
The antioxidant activity (FRAP) and anticancer activity (IC_50_) for fucoidans from the tested brown algae extracted via DM and UAE: *F. vesiculosus* (Fv), *F. distichus* (Fd), *F. serratus* (Fs), and *A. nodosum* (An). Values are expressed as the mean ± sd.

In the current study (Table 1, Figure 7), the most promising were fucoidans (<300 kDa) extracted via DM from *F. vesiculosus* and *F. serratus*. These two fucoidans presented the lowest IC_50_ value when compared with the other samples, especially those obtained using the UAE method.

The relationship between antioxidant activity and the anticancer efficacy of natural compounds has been the focus of numerous studies. In this work, we assessed the total antioxidant activity of fucoidan obtained from four various brown algae via two methods of extraction using the FRAP assay (Figure 7). Fucoidan from *F. vesiculosus* and *F. serratus*, obtained via DM showed the most prominent anticancer activity with an IC_50_ of 368 and 301 µg/mL, probably due to its high total phlorotannins content of about 5.4 and 5.1 %dw. The use of ultrasound resulted not only in a decrease in the level of TPC but also in a decrease in activity. Moreover, for the algae *F. vesiculosus*, *F. distichus*, and *A. nodosum*, the decrease in activity was 1.6–1.9 times, while for *F. serratus*, a decrease in activity by five times was observed. A significant reduction in TPC (by 73%) was also registered for *F. serratus* when DM was compared with UAE.

The results showed that fucoidan containing higher amounts of phlorotannins and lower levels of sulfates and uronic acid obtained via DM exhibited the highest total antioxidant activity. The same samples Fv1 and Fs1 demonstrated higher cytotoxic activity against HeLa G-63 cells than the other samples. This finding is especially noteworthy as it indicates that both the algae species and the extraction method considerably influence the activity of fucoidan. A strong positive correlation was observed between IC_50_ and Mw (Pearson correlation coefficient r = 0.95, *p* < 0.05). The interaction between method and species continued to be extremely significant (*p* < 0.0001), also when the two-way ANOVA analysis was applied to the data of the antioxidant and anticancer activities.

### 2.4. Study of Synergism

**Figure 8:**
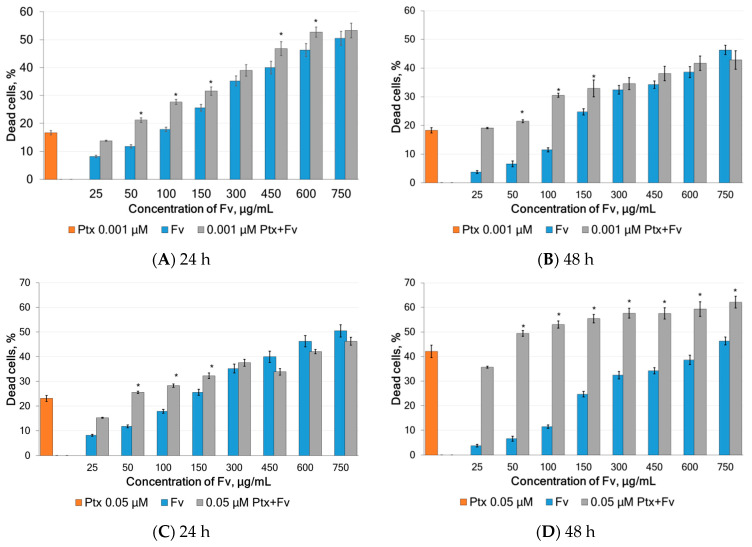
Inhibition of the viability of HeLa G-63 cells upon incubation with paclitaxel and different concentrations of fucoidan Fv alone or in combination for 24 and 48 h (*n* = 5). (**A**)—incubation with 0.001 µM Ptx and Fv for 24 h; (**B**)—incubation with 0.001 µM Ptx and Fv for 48 h; (**C**)—incubation with 0.05 µM Ptx and Fv for 24 h; (**D**)—incubation with 0.05 µM Ptx and Fv for 48 h. Results represent the mean ± sd from three independent experiments. * A significant difference from single treatments is indicated by *p* < 0.05.

Side effects restrain the application of chemotherapies in the clinic. Therefore, to improve drug efficacy, as well as reduce adverse effects, we tested whether fucoidan synergizes with Ptx in the treatment of cervical cancer. Based on the previously obtained IC_50_ values of fucoidan and Ptx, a dose-ranging experiment was designed and cell viability was determined in which fucoidan (25–750 μg/mL) and Ptx (0.001 and 0.05 μM) were added to HeLa G-63 cells at increasing doses either alone or in combination. The data are shown in Figure 8.

The exposition of HeLa G-63 cells to 0.001 µM Ptx together with fucoidan at 50–600 µg/mL for 24 h led to a significant (*p* < 0.05) increase in cell death (Figure 8A). When incubated for 48 h, significant increase in the anticancer effect was observed in the fucoidan concentrations from 50 to 150 µg/mL (Figure 8B).

Simultaneous application of Ptx at a concentration of 0.05 µM and fucoidan for 24 h on HeLa G-63 cells resulted in significant increase (*p* < 0.05) in dead cells at fucoidan concentrations of 50 and 150 µg/mL. Whereas, when incubated for 48 h, a statistically significant increase in the anticancer effect was observed in the fucoidan concentration range from 50 to 750 µg/mL (Figure 8C and Figure 8D, respectively).

**Figure 9:**
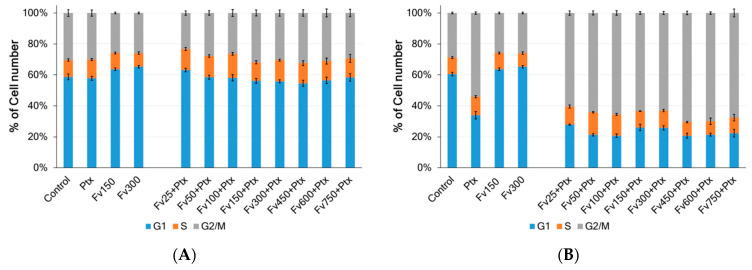
Distribution of HeLa G-63 cells by cell cycle phases under the combined action of paclitaxel (Ptx) (0.001 µM (**A**), 0.05 µM (**B**)) and different concentrations of fucoidan Fv after 48 h. The results are presented as mean ± sd from three separate experiments.

To investigate the mechanisms of the synergistic effect of fucoidan in combination with Ptx on HeLa G-63 cells, a propidium iodide (PI) staining assay using flow cytometry was used to analyze the cell cycle distribution (Figure 9). Cell cycle assays were performed after 48 h exposure of HeLa G-63 cells to different concentrations of Ptx (0.001 and 0.05 μM) and Fv (25–750 μg/mL) both separately and combined to test the hypothesis that the growth suppression has stemmed from the disruption in the cell (Figure 9).

Ptx affects the cell cycle, causing a delay in the G2/M phase. It has a cytotoxic antimitotic effect. When HeLa G63 cells were incubated with 0.001 µM Ptx as well as with its combined action with various concentrations of fucoidan, no changes in the distribution of cells throughout the cycle were observed (Figure 9A). Whereas when cells were incubated with 0.05 µM Ptx as well as with its combined action with various concentrations of 25–100 μg/mL fucoidan, an accumulation of cells in the G2/M phase was observed (Figure 9B). The percentage of cells in the G1 and G2/M phases changed significantly as a result of the combination treatment, suggesting that the combination of the two drugs caused cell death more effectively than either of them alone. This experiment showed that co-administration of Ptx (0.05 μM) and fucoidan from *F. vesiculosus* at low concentrations (25–100 μg/mL) caused cell cycle arrest in the G2/M phase.

Mathew et al. (2017) performed combination growth inhibition assays using the IC_50_ concentration of the chemotherapeutic agents, which included paclitaxel and tamoxifen, as well as 0.3 mg/mL fucoidan UPF and 1.3 mg/mL fucoidan FVF [58]. It was demonstrated that both compounds appeared to have overall synergistic activity in inhibiting cancer cell growth when used in combination with paclitaxel or tamoxifen. Our results are consistent with the work [59], which showed that the semisynthetic alginate derivative polymannuroguluronate sulfate (2 mg/mL) in combination with Ptx (50 nM) blocked the cell cycle at the G2/M phase in HeLa G-63 cells, similar to Ptx treatment alone. Moreover, the sulfated polysaccharide itself did not show activity.

**Figure 10:**
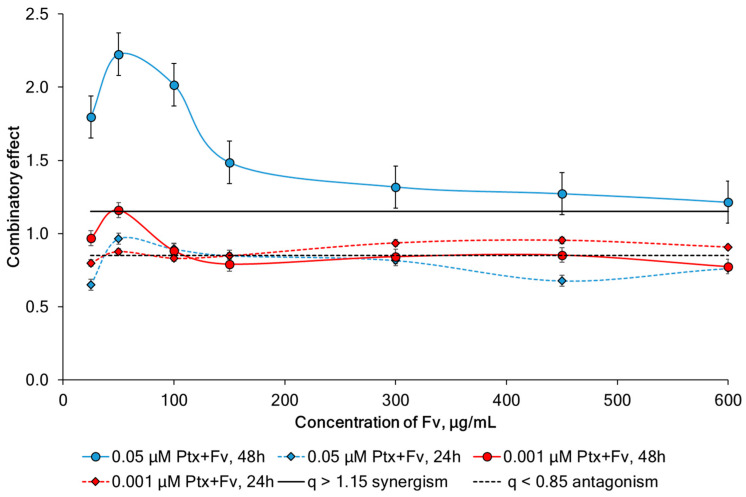
The combinatory effect (q) of paclitaxel (Ptx) and various concentrations of fucoidan (Fv) on HeLa G-63 cells.

The calculation of the combinatory effect (CE) showed that the simultaneous use of Ptx at a concentration of 0.05 μM and fucoidan at a concentration of 25–600 μg/mL after 48 h exposure leads to a synergistic interaction (Figure 10). At the same time, the combined action of the same mixtures after 24 h exposure does not give a synergistic effect. A decrease in the concentration of Ptx at the same concentrations of fucoidan also does not form synergistic combinations. Thus, synergism depends not only on the concentrations of the components but also on the time of exposure. However, our fucoidan from *F. vesiculosus* led to increased activity Ptx at a much lower concentration (25–100 μg/mL), and the additivity effect was observed even when using a Ptx concentration lower than 50 times the IC_50_ (0.001 μM) (Figure 10).

The anticancer activity of the fucoidan (100–400 μg/mL) extracted from *Nizamuddinia zanardinii* by UAE was lower than that extracted via enzyme and enzyme-UAE [60]. A previous report [61] showed that the anticancer activity of bioactive polysaccharides is attributable to various factors such as sulfate content, monosaccharide composition, molecular weight and amount of impurity of protein, and total phenol. Yang et al. (2008) proposed that the anticancer activities of brown algae fucoidans depend on species and growing conditions of algae, extraction and purification methods, and the cancer cell lines used in the tests [37]. The effects of different extraction technologies on the extraction of phytochemicals (fucoidan, sulfates, uronic acid, and TPC) from the tested samples of brown algae are presented in Table 1. There was a significant variation in the extraction of each phytochemical depending on the macroalgal species and technological treatments. In general, extracts from *F. vesiculosus* contained the highest levels of fucoidan and TPC compared to others. The differences in the recovery of phytochemicals between species could be attributed to inter-species differences, while the effect of technological treatments on all species was similar (Figure 2). Harnedy & FitzGerald (2013) mentioned that strong cell walls containing variable types and amounts of polysaccharides, depending on the macroalgal species, were one of the main obstacles hindering an efficient extraction of compounds from macroalgae [62]. Thus, the type of polysaccharides and ionic interactions with other cell wall constituents will be strongly influenced by the macroalgal species studied [63], affecting the yields of compounds extracted depending on the macroalgal species.

The cell cycle is a sequence of synchronized activities that leads to cell division. When cellular control over this normal proliferation is compromised, a normal cell becomes cancerous. Consequently, chemotherapeutic drugs may aim to induce cell cycle arrest. The DNA synthesis (S phase), mitotic (M phase), and two gap phases (G1, G2) are among the phases of the cell cycle. The G0 segment is the resting length inside the molecular cycle, wherein the cells stay inactive. The sub-G1 segment represents the proportion of apoptotic cells, so the molecular cycle evaluation can offer apoptotic data. The G1 segment is, as a substitute, vital, considering the fact that arrest at this level affords a possibility for cells to both go through restore mechanisms or comply with the apoptotic pathway. Therefore, most of the anticancer drugs target this stage of the cell cycle [64]. Ptx + Fv showed a prominent cell cycle arrest at the G1 phase and accumulation of the cells in the S phase. The data indicate that the anticancer potential of ESPs-CP is mainly attributed to the induction of apoptosis and cell cycle arrest at the G1 phase. The anticancer activity of sulfated polysaccharides from brown alga *Ecklonia cava* [64], *Sargassum horneri* [65] and *Padina tetrastromatica* [66] also claimed the same pattern of cell cycle arrest.

The lack of selective targeting of chemotherapeutic agents such as Ptx leads to the high toxicity of therapy and the incidence of serious side effects, which not only reduces the quality of life but can also cause serious complications [67]. Consequently, there is an interest in agents that could either enhance the beneficial effects of conventional therapy or reduce the incidence of side effects. To date, several agents effective in enhancing the response to Ptx have emerged and include various flavonoids [68], green tea extract [69], etc. In the current study, we observed that co-administration of fucoidan to Ptx-treated cells enhanced the therapeutic effect in the tested cell line. In addition, subsequent analysis of the combined effect of the drug revealed synergy between fucoidan and Ptx. Similar results were obtained by Zhang et al. (2013), who investigated the effects of a common cytostatic combination with fucoidans [70]. The authors reported that the combined use of cytostatics and fucoidan provided a greater therapeutic effect compared to treatment with a single agent. They also showed that the combination of fucoidan and cisplatin was the most effective. Abudabbus et al. (2017) have suggested that co-incubation of cisplatin, doxorubicin, or Ptx with fucoidan enhanced the therapeutic effects compared to individual agents [71]; however, synergy was not calculated. The synergy between fucoidan and lapatinib [72] and fucoidan and cisplatin on HNSCC cell lines (H103, FaDu, and KB) was also reported [73].

This result may be explained by the multifactorial nature of the anticancer mechanisms of natural compounds. Although antioxidants can neutralize reactive oxygen species and decrease oxidative damage, which might also additionally save you from most cancers, the induction of apoptosis in most cancer cells regularly entails extra mechanisms inclusive of the modulation of molecular signaling pathways and induction of endoplasmic reticulum stress [74]. In this scenario, fucoidan derived from *F. vesiculosus*, exhibiting the most significant antioxidant properties and the highest level of cytotoxicity, could operate via several mechanisms to trigger cell death in HeLa G-63 cells. Studies of the synergistic effects of antioxidants with anticancer drugs may provide a promising basis for designing new agents for cancer therapy.

## 3. Materials and Methods

### 3.1. Sample Collection and Preparation of Alga

The brown alga, bladder wrack (*Fucus vesiculosus* L.) (Fv), toothed wrack (*Fucus serratus* L.) (Fs), rockweed (*Fucus distichus* L.) (Fd), and Icelandic kelp (*Ascophyllum nodosum* L.) Le Jolis) (An) were collected in August–September 2023 from the littoral of the Barents Sea (Zelentskaya Bay, Murmansk region, Russia) at low tide. Dr. E. Obluchinskaya identified the algae, and the voucher specimen (No. 9.2023) was deposited in the Collection of the Zoobentos Laboratory, Murmansk Marine Biological Institute. Collected algae were thoroughly washed in running tap water, followed by distilled water, to remove the adhering sand, epiphytes, extraneous matter, and necrotic parts, frozen within two hours of collection and subsequently freeze-dried for one week to ensure full lyophilization, and ground to a fine powder. Dried samples were stored in sealed containers at 4 °C until use.

### 3.2. Materials

Folin–Ciocalteu reagent, Trolox (6-hydroxy-2,5,7,8-tetramethylchroman-2-carboxylic acid), TTTZ (2,4,6-tripyridyl-s-triazine), ascorbic acid, phloroglucinol, L-cysteine, ammonium molybdate, analytical grade monosaccharides (fucose (Fuc), xylose (Xyl), mannose (Man), galactose (Gal), arabinose (Ara) and glucose (Glc), all purities > 99%), Shodex pullulan P-82 standard kit (Showa-Denko Co., Tokyo, Japan) and blue dextran-2000 (GE Healthcare, Chicago, IL, USA) were obtained from Sigma-Aldrich (St. Louis, MO, USA). Versene solution, phosphate-buffered saline (PBS), and Dulbecco’s modified Eagle medium (DMEM) supplemented with 10% heat-inactivated fetal bovine serum (FBS) and 100 IU/mL penicillin and streptomycin were from BioloT Ltd., St. Petersburg, Russia. Propidium iodide (PI) and 3-(4,5-dimethylthiazole-2yl)-2,5-diphenyl tetrazolium bromide (MTT) were purchased from LLC Company Pushchino Laboratories, Pushchino, Russia. An aqueous solution of paclitaxel (Sandoz Pharmaceuticals d.d., Ljubljana, Slovenia) at a concentration of 6 mg/mL was stored at room temperature in the dark for future analysis. Ultrapure water was purified using a Milli-Q system (Millipore, Bedford, MA, USA). Other analytical grade chemicals and solvents for extraction and analysis were of analytical grade and purchased from local chemical suppliers.

### 3.3. Fucoidan Extraction

Fucoidans were prepared using a method modified from [49]. Briefly, samples of dried algae were pretreated sequentially with an azeotrope (methylene chloride/EtOH) and 80% EtOH in a Soxhlet apparatus to remove lipids, pigments, and mannitol. The defatted algae were extracted with 5% (*v*/*v*) EtOH at pH 4, and dry algae: liquid ratio of 1:10 (*w*/*v*). Fucoidan was isolated using UAE and DM. Residual supernatant and algae were separated. Alginate was removed by precipitation with 2% NaHCO_3_ and centrifugation. The supernatant was concentrated using AR-3–100PS cassettes (NTP Biotest, Kirishi, Russia). After purification, the solution was lyophilized for 24 h on an Inei-4 freeze dryer (IBF RAS, Pushchino, Russia). The yield of fucoidan (g/100 g dw) was calculated using the following Equation (1):

Yield Fucoidan = [dry wt. of fucoidan/dry wt. of algae] × 100(1)

#### 3.3.1. Dynamic Maceration (DM)

Samples of defatted algae were twice macerated on a magnetic stirring apparatus equipped with a temperature-controlling system at 200 rpm with heating at 60 °C with the same solvent. All the other steps were equally performed in both UAE and DM (Fv1, Fd1, Fs1, and An1).

#### 3.3.2. Ultrasonic-Assisted Extraction (UAE)

An ultrasonic processor (UZTA-0.4/22-OM, Biysk, Russia) with a 15 mm probe was used. Samples of defatted algae with a solvent were twice sonicated (22 kHz) at room temperature for 20 min (Fv2, Fd2, Fs2, and An2).

### 3.4. Analysis of Fucoidan Composition

The fucoidan content was determined using the method [75]. Fucoidan content was measured via the cysteine–sulfuric acid method using L-fucose as a reference [76]. All measurements were done in duplicate, and results were expressed as the percentage of fucose in dry fucoidan. The sulfate residues were determined using the BaCl_2_-gelatin method [77]. Uronic acid was determined following the Scott method (1979) with 3,5-dimethylphenol as the reagent [78] and alginic acid as a standard [79]. Impurities of polyphenols, as the total phlorotannin content (TPC), were quantified using Folin–Ciocalteau reagent with phloroglucinol as the standard [80], and the results were expressed as g of phloroglucinol equivalent per 100 g (%) of fucoidan DW.

The molecular weight (Mw) of fucoidan was analyzed using high-performance size-exclusion chromatography (HPSEC) [81], equipped with two TSKGel columns (Tosoh Bioscience, Stuttgart, Germany) and a PWX guard column. Water was used as the mobile phase at a rate of 0.4 mL/min at 70 °C. The monosaccharide compositions of fucoidans were analyzed using a high-performance liquid chromatography system (HPLC Model LC 20 AT Prominence, Shimadzu, Kyoto, Japan) with a refractive index detector (RID-10A, Shimadzu, Japan) after hydrolysis [82]. The samples were injected into a Shodex Asahipak NH2P-50 4E (4.6 × mm, 5 µm) column (Kanagawa, Japan) at 50 °C under isocratic conditions with a mobile phase consisting of 0.25 M H_3_PO_4_-acetonitrile (20:80, *v*/*v*) at a flow rate of 1.0 mL/min. Data are presented as relative mole percentages.

### 3.5. In Vitro Antioxidant Assays

Ferric reducing antioxidant power (FRAP) assay was performed following the method [83]. FRAP results were expressed as μM of Trolox equivalents (μM_TE_).

### 3.6. Cell Culture

Human carcinoma HeLa G-63 cells were obtained from the shared research facility of Vertebrate Cell Culture Collection (Institute of Cytology RAS, St. Petersburg, Russia) and have been cultivated in the laboratory of PNPI since 1963.

After cryopreservation at −80 °C, thawed HeLa G-63 cells (1.5 × 10^6^ cells/mL) were seeded in 60 mm culture dishes in 4 mL of culture medium. Cells were incubated at 37 °C in a humidified atmosphere with 5% CO_2_, and the culture medium was changed every 24 h. For cell culture, cells were detached from the 60 mm culture dish using 0.25% trypsin: 0.02% Versene solution (1:3 *v*/*v*) and replated on a new culture dish. After 3–4 days, cells attached and spread on the 60 mm culture dishes and were subcultured to 80–90% confluence. Passages were repeated three to five times, the optimal density was 1.0–5.0 × 10^4^ cells/cm^2^. The HeLa G-63 was cultivated in monolayer culture in DMEM with L-glutamine and with 4.5 g/L glucose, and supplemented with 10% heat-inactivated FBS and 100 IU/mL penicillin and 100 μg/mL streptomycin in an incubator with 5% CO_2_ at 37 °C.

#### 3.6.1. Proliferation Assay and Cell Cycle Analysis

Cells were seeded at a density of 2 × 10^5^ cells/well in 12-well plates and incubated for 24 h at 37 °C in a CO_2_ incubator to form a monolayer. Then, the cells were treated with different concentrations of fucoidans and a reference drug for 24 h. After treatment, the cells were separated using Versene solution, washed with PBS, centrifuged (1000 rpm for 5 min), and the resulting suspension was stained with PI solution containing Rnase [84]. The antiproliferation activity and cell cycle analysis were carried out using a CytoFLEX B3-R2-V2 flow cytometer (Beckman Coulter, Brea, CA, USA; 20,000 cells per experiment) [85].

#### 3.6.2. Cytotoxicity Assay

The cytotoxicity of the samples was determined via MTT assay [86]. In brief, HeLa G-63 cells were plated at 2 × 10^5^ cells per well in 96-well microtiter plates (Cell+, Sarstedt, Germany) with 100 mL RPMI-1640 growth medium and incubated for 24 h at 37 °C, with 5% CO_2_ in a humidified atmosphere. Thereafter, the medium was removed and fresh serum-free medium containing different concentrations (25, 50, 100, 150, 300, 450, 600 and 750 μg/mL) of the tested fucoidans and chemotherapeutic agents at a log range of concentrations (Ptx, 1 nM–800 µM) was added. After 44 h of incubation, the 10 μL MTT reagent (5 mg/mL) was added. After incubating at 37 °C for 2 h, the MTT reagent was removed, dimethyl sulphoxide (DMSO; 100 μL) was added to each well and thoroughly mixed by pipetting.

The absorbance was measured using a plate reader Multiskan FC (Thermo Scientific, Waltham, MA, USA) at 570 nm, and the percent cell death was calculated. Negative control cells were incubated without compounds. The percentage inhibition of proliferation was calculated using the formula below, and the half maximal inhibitory concentration of tested samples (IC_50_) was calculated from the dose-response [87].

Cell Viability (%) = 100% (Test Concentration Reading − Blank Reading)/(Control Reading − Blank Reading) (2)

#### 3.6.3. Analysis of Drug Combinatory Effect

The cytotoxicity of the combined use of fucoidan Fv and Ptx was assessed using flow cytometry in combinations of a constant concentration of Ptx with increasing concentrations of fucoidan. In the combined experiments, cells were incubated in 12-well plates and treated with Fv (500 μL) and Ptx (500 μL). After the incubation, the cells were removed with Versene solution, spun down, and the resulting suspension was stained with PI solution (50 μL/mL) for 20 min at 37 °C. The modified probability sum test [88] was applied to assess the mode of combinatory effect, according to the equation:

q = P[A + B]/(P[A] + P[B] − P[A] × P[B])(3)

where P[A] represents the efficacy of drug A and P[B] the efficacy of drug B, while P[A + B] depicts the efficacy of a combination of both drugs at the same doses. Assessed value/expected value (sum of probability of independent events) ratios were defined: q < 0.85 as antagonism, while q = 1.00 ± 0.15 indicates additivity, and q > 1.15 synergism.

### 3.7. Statistical Analysis

Each experiment was repeated three times. Data are represented by mean ± standard deviation (±sd). Statistical analysis was performed with STATGRAPHICS Centurion XV (StatPoint Technologies Inc., Warrenton, VA, USA).

## 4. Conclusions

The effect of the extraction method (dynamic maceration and ultrasound-assisted extraction) on the composition and properties of fucoidans from Arctic brown algae *F. vesiculosus*, *F. serratus*, *F. distichus*, and *A. nodosum* was explored. It was found that fucoidans containing higher TPC and lower levels of sulfates and uronic acid obtained via dynamic maceration exhibited the highest antioxidant and anticancer activity compared with fucoidans obtained via the UAE. For the first time, the synergy for a binary combination of 0.05 µM Ptx with 50–750 µg/mL Fv against HeLa G-63 cell growth was calculated. Thus, our results support the rationality of fucoidan use as a powerful antioxidant and in complex chemotherapy.

Our results open new perspectives and challenges. Future kinetic modeling of fucoidan degradation and more advanced characterization techniques could offer a deeper understanding of structural–function relationships. Our new data provide a reliable basis for the future validation of established synergy through additional mechanistic studies, such as apoptosis pathway analysis and gene expression profiling.

## Figures and Tables

**Table 1:** Yield and chemical composition of fucoidans isolated from Arctic brown algae.

Species	Extraction Method	Sample	Fucoidan Yield, %dw	Fucoidan, %dw	Mw, kDa	IC_50_, μg/mL	TPC, %dw	FRAP, μM_TE_
*F. vesiculosus*	DM	Fv1	9.4 ± 0.4 *	59.4 ± 0.2 *	244 ± 8 *	368 ± 13 *	5.4 ± 0.1 *	185 ± 4 *
	UAE	Fv2	17.4 ± 0.3	64.1 ± 0.4	195 ± 11	640 ± 12	3.3 ± 0.1	159 ± 5
*F. distichus*	DM	Fd1	11.2 ± 0.3 *	58.8 ± 0.3 *	273 ± 7 *	626 ± 20 *	4.2 ± 0.2 *	175 ± 5 *
	UAE	Fd2	14.9 ± 0.7	63.5 ± 0.2	229 ± 9	1212 ± 34	1.6 ± 0.1	96 ± 6
*F. serratus*	DM	Fs1	8.0 ± 0.3 *	53.1 ± 0.2 *	263 ± 8 *	301 ± 17 *	5.1 ± 0.3 *	176 ± 6 *
	UAE	Fs2	12.6 ± 0.5	57.7 ± 0.5	239 ± 6	1498 ± 54	1.4 ± 0.2	94 ± 4
*A. nodosum*	DM	An1	12.4 ± 0.4 *	58.8 ± 0.6 *	364 ± 10 *	667 ± 20 *	6.7 ± 0.3 *	194 ± 9 *
	UAE	An2	13.8 ± 0.3	60.7 ± 0.1	222 ± 7	1042 ± 30	3.6 ± 0.4	156 ± 6

Data is shown as a mean ± sd (*n* = 3). * Multiple comparison analyses show statistically significant differences (*p* < 0.05) between DM and UAE extraction methods. Mw—molecular weight; IC_50_—anticancer activity; TPC—total phlorotannins content; FRAP—Ferric reducing antioxidant power.

**Table 2:** Structural units of fucoidans vs. method extraction.

Sample	Monosaccharide Composition, mol.%					Uronic Acid, %	Sulfate, %
	Fucose	Xylose	Mannose	Glucose	Galactose		
Fv1	71.9 ± 0.9 *	9.4 ± 0.4	6.0 ± 0.2	8.3 ± 0.3	4.4 ± 0.1 *	5.3 ± 0.4 *	25.4 ± 0.3 *
Fv2	75.7 ± 1.1	7.6 ± 0.5	4.3 ± 0.1	5.8 ± 0.4	6.6 ± 0.1	6.4 ± 0.3	23.8 ± 0.2
Fd1	63.6 ± 1.2 *	4.2 ± 0.3	4.8 ± 0.2	21.6 ± 0.7	5.8 ± 0.2 *	5.9 ± 0.5 *	26.6 ± 0.5 *
Fd2	68.2 ± 0.8	5.8 ± 0.3	4.7 ± 0.2	17.9 ± 0.9	3.4 ± 0.1	7.0 ± 0.5	25.4 ± 0.3
Fs1	69.7 ± 0.7 *	5.9 ± 0.5	8.1 ± 0.3	3.0 ± 0.2	13.3 ± 0.3 *	4.1 ± 0.4 *	22.4 ± 0.4 *
Fs2	72.8 ± 1.0	4.5 ± 0.4	7.5 ± 0.2	4.4 ± 0.3	10.8 ± 0.4	4.8 ± 0.2	20.9 ± 0.2
An1	47.8 ± 1.2 *	12.4 ± 0.3	14.3 ± 0.4	13.3 ± 0.4	12.2 ± 0.5	3.3 ± 0.6 *	17.6 ± 0.2 *
An2	49.3 ± 0.9	11.7 ± 0.5	14.0 ± 0.5	13.4 ± 0.3	11.6 ± 0.4	4.5 ± 0.5	16.4 ± 0.4

Data is shown as a mean ± sd (*n* = 3). * Multiple comparison analyses show statistically significant differences (*p* < 0.05) between DM and UAE extraction methods.

## Data Availability

Data are contained within the article.
